# Non-Transfusion-Dependent Thalassemia: An Update on Complications and Management

**DOI:** 10.3390/ijms19010182

**Published:** 2018-01-08

**Authors:** Joseph Sleiman, Ali Tarhini, Rayan Bou-Fakhredin, Antoine N. Saliba, Maria Domenica Cappellini, Ali T. Taher

**Affiliations:** 1 Department of Internal Medicine, American University of Beirut Medical Center, Beirut 11-0236, Lebanon; joseph.sleiman.22@gmail.com (J.S.); ali.ht13@gmail.com (A.T.); rib05@mail.aub.edu (R.B.-F.); 2 Department of Medicine, Indiana University School of Medicine, Indianapolis, IN 46202, USA; antoine.nabil.saliba@gmail.com; 3 Department of Medicine, Ca’Granda Foundation IRCCS, University of Milan, 20122 Milan, Italy; maria.cappellini@unimi.it; 4 Department of Clinical Science and Community, University of Milan, 20122 Milan, Italy

**Keywords:** non-transfusion dependent thalassemia, morbidity, management, ineffective erythropoiesis, iron overload, iron chelation therapy

## Abstract

Patients with non-transfusion-dependent thalassemia (NTDT) experience many clinical complications despite their independence from frequent transfusions. Morbidities in NTDT stem from the interaction of multiple pathophysiological factors: ineffective erythropoiesis, iron overload (IOL), and hypercoagulability. Ineffective erythropoiesis and hemolysis are associated with chronic hypoxia and a hypercoagulable state. The latter are linked to a high prevalence of thromboembolic and cerebrovascular events, as well as leg ulcers and pulmonary hypertension. IOL in NTDT patients is a cumulative process that can lead to several iron-related morbidities in the liver (liver fibrosis), kidneys, endocrine glands (endocrinopathies), and vascular system (vascular disease). This review sheds light on the pathophysiology underlying morbidities associated with NTDT and summarizes the mainstays of treatment and some of the possible future therapeutic interventions.

## 1. Introduction

The group of disorders referred to as the thalassemias is one of the most common monogenic diseases worldwide. Having been historically clustered in the Mediterranean, North Africa, and South Asia, thalassemias are now encountered in other regions around the globe with the more recent immigration movements towards areas of lower prevalence [1,2]. Thalassemias result from an inherited imbalance between *α* and *β* chains of hemoglobin, instigating ineffective erythropoiesis [3].

Non-transfusion dependent thalassemia (NTDT) is a group of thalassemic disorders including patients who do not require frequent blood transfusions for survival. Those patients have Hemoglobin H disease or reduced expressivity of the *β* genes (homozygous *β*^+^ or compound heterozygous *β*^+^/*β*^0^ in addition to other variants where the end result remains *α*/*β* imbalance (Table 1) [4]. Patients with NTDT may still require occasional or more frequent red blood cell (RBC) transfusion therapy in certain circumstances including but not limited to significant infection, pregnancy, periods of rapid growth, or surgery [2,5]. Over the years, many individual studies have showed a clear variation in the complications seen between transfusion dependent (TDT) and non-transfusion dependent (NTDT) thalassemic patients. The difference in management (i.e., limited transfusion, limited chelation, more frequent splenectomies) is an attributable factor in such discrepancy in the multimorbidity profiles of TDT and NTDT [6,7,8]. Ultimately, the OPTIMAL CARE study was one of the first landmark studies that confirmed the high prevalence of unique morbidities in patients with *β*-thalassemia intermedia (TI) as compared to *β*-thalassemia major (TM) [9]. The need to consider the NTDT patients as a separate population, in terms of guidelines for complications screening and management, has since been acknowledged. This review describes the underlying pathophysiology that separates NTDT from TDT, and delineates the various morbidities associated with this subgroup of thalassemia while comparing it to TDT. Discussion of current and prospective options for management follows.

## 2. Pathophysiology

Both TDT and NTDT share the pathological cascade of *α*-to-*β* globin chain imbalance, ineffective erythropoiesis, and an array of subsequent pathophysiological mechanisms underlying the morbidity profiles [10]. While transfusion therapy helps suppress ineffective erythropoiesis, it is also associated with secondary complications [11]. Since current therapeutic strategies for NTDT are primarily initiated when symptoms or complications occur, the clinical picture of NTDT patients, as compared to TDT patients, is predominated by the long-term effects of chronic anemia and tissue hypoxia and their compensatory reactions, including bone marrow expansion, ineffective erythropoiesis, and increased intestinal iron absorption [12,13].

### 2.1. Ineffective Erythropoiesis

*α*- and *β*-globin chain imbalance leads to a chronic state of anemia and ineffective erythropoiesis [5]. In *β*-thalassemias, for example, the formation of hemichromes from excess *α*-globin chains stimulates an oxidative stress that is able to damage membranes of both mature and immature erythroid cells [14]. The increase in erythropoietin driven by anemia and the consequent cascade of Janus Kinase 2 (JAK2)-dependent phosphorylation events ultimately expand the erythroid lineage within the bone marrow. This expansion, however, fails to produce enough mature RBCs in the peripheral blood, and rather results in characteristic bone deformities and formation of hematopoietic pseudotumors and/or hepatosplenomegaly [15].

### 2.2. Chronic Hemolytic Anemia

Both intravascular and extravascular hemolysis can occur. Lower hemoglobin levels in patients with NTDT are associated with higher prevalence of complications [16]. It is thought that the exposure to senescence antigens during ineffective erythropoiesis, chronic platelet activation, and increased platelet aggregation lead to a hypercoagulable state in NTDT [17]. Chronic hemolytic anemia is associated with chronic hypoxia, generation of reactive oxygen species, and dysregulation of the hepcidin/iron homeostasis in favor of increased iron absorption. Both anemia and iron overload can further worsen ineffective erythropoiesis and complicate the pathophysiological picture [18].

### 2.3. Iron Overload

No active mechanism for excess iron excretion exists in the human body. The mechanism of iron overload in TDT and NTDT is distinct in many aspects. Complications associated with iron overload appear much earlier in patients with TDT as compared to patients with NTDT [19]. Ineffective erythropoiesis plays a central role in the process in patients with NTDT and results in significant iron overload despite absent or minimal transfusional iron burden. Ineffective erythropoiesis triggers suppression of hepcidin synthesis leading to the upregulation of intestinal iron absorption, and iron release from macrophages [20]. Twisted gastrulation factor-1, hypoxia inducible transcription factors, transmembrane protease serine-6 (TMPRSS-6), erythroferrone, and possibly growth differentiation factor-15 have been suggested to play a role in hepcidin regulation [21,22,23,24]. Although the exact mechanisms are still incompletely understood, the common end point is an increased iron availability and release in the circulation with subsequent end-organ damage to the liver, endocrine glands, myocardium, and others.

## 3. Morbidities in NTDT

**Figure 1:**
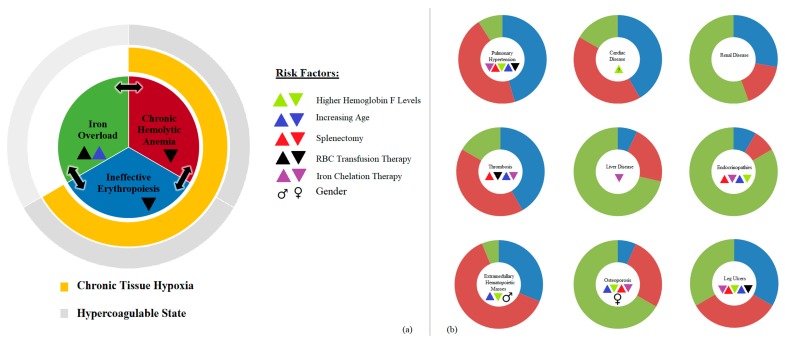
(**a**) Pathophysiology related to NTDT, and (**b**) qualitative representation of interplay between NTDT morbidities and pathophysiology. Some independent risk factors that were studied were also added.

Figure 1 depicts the interplay between NTDT morbidities and pathophysiology.

### 3.1. Thrombosis

The hypercoagulable state in NTDT is associated with a high frequency of thromboembolic complications, such as deep vein thrombosis, portal vein thrombosis, pulmonary thromboembolism, cerebral thrombosis, and recurrent arterial thrombosis. Thromboembolic events are more highly prevalent in patients with NTDT as compared to patients with well-transfused TDT; splenectomized patients with NTDT were at a significantly higher risk for thromboembolic events [25,26]. Independent risk factors are splenectomy, serum ferritin level ≥ 1000 ng/mL, hemoglobin level < 9 g/dL, and age > 35 years [9,26,27]. In splenectomized NTDT patients, nucleated RBC count > 300 × 10^6^/L, platelet > 500 × 10^9^/L, and RBC transfusion naivety were associated with higher prevalence of thromboembolic disease [27].

Ischemic strokes have been reported in combination with thromboembolic disease and linked to cardiac valvular lesions (elastic tissue defects) or atrial fibrillation [28]. Arterial stenosis on magnetic resonance angiography was found in 27.6% of 29 asymptomatic splenectomized NTDT adults, most commonly occurring in the internal carotid artery [29].

Simultaneously, silent thrombosis can occur, as subclinical thrombi in microvasculature of lungs and brain have been described on autopsy in the absence of other known risk factors [30]. Silent cerebral infarcts rate in TI patients was estimated at 27–60%, and white matter lesions frequency correlated with age, transfusion naivety, and splenectomy [31]. Higher liver iron concentration (LIC) values correlated with abnormal findings on positron emission tomography-computer tomography studies [32]. The clinical significance of these findings and the role of iron overload as an independent risk factor are to be clarified.

### 3.2. Cardiac Disease

Cardiac disease is the major cause of death in both TDT and NTDT populations [33,34,35]. While the main finding in TDT was iron overload leading to left ventricular (LV) dysfunction, cardiac failure, and cardiogenic death, most cardiac disease in NTDT relates to chronic right heart failure, secondary to pulmonary hypertension (PHT) [12,35]. Iron deposition in the heart is more prevalent and happens at a faster rate in TDT patients as compared to NTDT patients [19,36,37]. Older NTDT patients may still be prone to LV decompensation [13,34]. Several multicentric cross-sectional studies showed a higher prevalence of rhythm disorders, pericardial disease, and valvular abnormalities in NTDT as compared to healthy controls. However, high cardiac output (CO), increased pulmonary vascular resistance (PVR), and PHT were the most significant cardiac findings, especially when compared to TDT [12,33,35]. Increased CO stems from chronic anemia/hypoxia and related shunt development due to increased intramedullary and extramedullary erythropoiesis. HbF’s increased oxygen affinity and the dilatation of blood vessels secondary to coexistent elastic tissue injury, may also contribute to the increased CO [13,38]. Increased PVR has a multifactorial origin: besides endothelial injury from high CO, NTDT patients suffer from recurrent respiratory tract infections, chest wall deformities, extramedullary hematopoietic masses (that may be intrapulmonary), and age-related diffuse elastic disorders [38]. Iron overload may play an added role, given the known impact of hemosiderosis on pulmonary fibrosis. However, with universally lower ferritin level in NTDT and appropriate iron chelation therapy practices when needed, this remains a minor factor [39].

### 3.3. Pulmonary Hypertension

PHT is a complication of disease progression in the absence of transfusion therapy [12]. Anecdotally, a study reported PHT in 66% of TDT patients who had inadequate transfusion management, reinforcing the impact of long-term hypoxia [40]. Hypercoagulability, in the form of chronic thromboembolic disease, has also been implicated in the pathophysiology, as autopsies revealed extensive pulmonary arterial lesions in thalassemic patients who underwent splenectomy [28]. The prevalence of PHT was found to be 21.5% with groups of age 17.05 ± 5.8 years, but can reach 64% with older groups [34,41]. NTDT patients (4.8%) are 5 times more likely to have PHT than TDT (1.1%) patients. Risk factors were determined to be splenectomy, naivety to iron chelation therapy, naivety to hydroxyurea treatment, naivety to RBC transfusion therapy, a nucleated RBC count greater than 300 × 10^6^/L, a history of previous thromboembolic events, and older age [42,43]. The negative effect of hemolysis on nitric oxide and arginine availability has been heavily implicated in this phenomenon [44]. Annual routine echocardiography assessing tricuspid valve jet velocity is recommended, with a threshold of 3.2 m/s indicating a positive predictive value of 93.3% [42,43]. Current guidelines for PHT management with a cardiologist are used in NTDT, as more studies are needed for this specific population [11].

### 3.4. Leg Ulcers

NTDT patients have a higher risk of developing leg ulcers compared to the regularly transfused TDT patients [45]. Increased incidence in NTDT patients has been noticed especially in poorly controlled disease. Their typical location is at the medial and lateral malleoli, and they are mostly seen during the second decade of life, with increasing risk with age [46,47]. Being recurrent and slow to heal, leg ulcers are associated with significant morbidity by causing pain and disability [48]. Pathogenesis of leg ulcers is the result of interplay of many factors, notably chronic anemia and hypercoagulability. In addition, elevated venous pressure due to liver injury or right heart failure and RBC membrane defects and rigidity contribute to poor tissue oxygenation and render the skin injury-prone to minimal trauma [46,49]. No clear-cut recommendations exist for management of leg ulcers. Although previously theorized that the oxygen-retaining capacity of HbF further contributes to incidence of leg ulcers, increased HbF levels was found to be protective from leg ulcers [5,9,50]. Additionally, iron chelation and blood transfusions are beneficial with no clearly defined guidelines. Topical antibiotics, occlusive dressing, and leg elevation are helpful conservative measures [5,49].

### 3.5. Hepatobiliary Complications

As the majority of iron accumulation targets the liver in NTDT, patients are at increased risk of developing liver fibrosis, cirrhosis, and eventually hepatocellular carcinoma (HCC), mainly in non-chelated patients [51]. HCC prevalence is higher in NTDT as compared to TDT patients [52]. Incidence of HCC in thalassemia patients has been increasing with time. Iron overload is the single most important risk factor in patients who do not have chronic hepatitis C (HCV). Iron overload is associated with the formation of toxic free radicals and damages tumor suppressor genes and DNA repair genes. Additionally, iron overload accelerates the process of liver cirrhosis via its profibrogenic effects. It is important to recognize that iron overload may be associated with HCC even in the absence of liver cirrhosis [52,53]. HCV infection is a second key factor and appears to work in synergy with iron overload to increase risk of HCC development. On the other hand, hepatitis B virus (HBV) infection, despite its correlation with half HCC cases worldwide, has no established role in hepatic carcinogenesis in thalassemia patients [51].

Screening is necessary for early detection and treatment given the absence of symptoms in 82% of patients with NTDT affected by HCC. Close Surveillance of iron overload via non-invasive quantification of LIC with R2 or R2* MRI is favored over the older invasive liver biopsy [54]. However, the widely available and inexpensive method of serum ferritin measurement remains the most heavily used method, especially in resource-poor areas where MRI is unavailable, despite frequent underestimation of the actual iron burden in NTDT patients [55,56]. Additionally, NTDT patients with HCV infection, HBV infection, serum ferritin ≥ 1000 ng/mL, LIC ≥ 5 mg Fe/g dry weight (dw), or advanced cirrhosis, are recommended to undergo biannual hepatic ultrasound assessment for HCC screening.

Chronic hemolytic anemia in NTDT leads to formation of gallstones. Symptomatic gallstones should be treated with cholecystectomy. In addition, in the absence of symptoms of cholelithiasis, the gallbladder should be inspected during splenectomy and intervention should be considered as splenectomized patients are at a high risk should cholecystitis develop [57].

### 3.6. Extramedullary Hematopoiesis

Reactivation of dormant hematopoietic sites from fetal life is common during states of chronic ineffective erythropoiesis [58]. These findings parallel the fact that extramedullary hematopoietic masses (EHM) occur almost exclusively in NTDT patients compared to TDT (20% vs. <1%). EHMs in TDT occur when transfusion is inadequate, a quasi-NTDT state [8,9]. Physiologically speaking, the use of regular blood transfusions can decrease the need for extramedullary hematopoiesis; thus resulting in relative inactivity of these tissues, and leading to the shrinkage of any possible mass. Expansion of hematopoietic tissue in response to ineffective erythropoiesis can involve the reticuloendothelial system, resulting in pseudotumors in the liver, spleen, and other sites [58]. Risk factors for extramedullary hematopoietic tumors include male sex, older age, and lower HbF levels [10,19,50]. In clinical practice, more attention is allocated to paraspinal extramedullary hematopoieses, which account for 11–15% of EHMs and put patients at risk of spinal cord compression [59]. Management for symptomatic spinal masses can range from transfusion and hydroxyurea therapy to radiation and surgical decompression [5,11,58].

### 3.7. Bone Disease

Bone abnormalities, including facial bone deformities, protrusion of the upper jaw, obliteration of maxillary sinuses, and osteoporosis, appear to be more marked in NTDT as compared to TDT as a result of the enhanced ineffective erythropoiesis and consequent bone marrow expansion [60]. Patients with NTDT are at higher risk of osteoporosis with splenectomy, iron overload, low fetal hemoglobin levels, and female gender [9,61,62]. Lower rates of osteoporosis are observed in patients on iron chelation therapy or hydroxyurea therapy [9]. The most recent guidelines by the Thalassemia International Federation (TIF) recommend that all patients ≥10 years of age be screened by yearly assessment of lumbar spine, femoral neck, and distal ulna bone mineral density (BMD) via Dual Energy X-ray absorptiometry (DEXA) [11]. Calcium and vitamin D supplementation is frequently offered although its efficacy has not been fully established [63]. Bisphosphonates remain the gold standard of thalassemia-associated osteoporosis treatment in both TDT and NTDT [63,64].

### 3.8. Endocrinopathies/Delayed Growth

It is important for the clinician to recognize endocrine disease as an important complication of NTDT [65]. When compared to patients with TDT, the lower prevalence of endocrine disease may be attributed to the lower extent, slower rate, and hepatic predominance of iron loading in patients with NTDT [66]. Iron accumulation results in dysregulation of the hypothalamic-pituitary axis and results in multiple endocrine disorders, including hypogonadism and delayed puberty. Splenectomy is identified as a prominent risk factor for hypothyroidism [67].

The prevalence of endocrinopathies—such as hypogonadism, hypothyroidism, hypoparathyroidism, diabetes mellitus, and adrenal insufficiency—in NTDT increases with age [19]. Iron chelation therapy and hydroxyurea therapy have been associated with a lower frequency of endocrine complications [9]. Fertility is not severely affected and patients can still achieve pregnancy spontaneously; however, pregnancy in this patient population is complicated by high risk of abortion, thromboembolic events, and intrauterine growth restriction in more than half of cases [68]. It is recommended to run the following tests annually in all NTDT patients ≥10 years: calcium, phosphate, 25-hydroxyvitamin D, free thyroxine, thyroid stimulating hormone, fasting plasma glucose, and adrenocorticotropic hormone stimulation test [65].

### 3.9. Renal Disease

Chronic anemia and hypoxia induce activation of fibroblasts and damage to tubular and endothelial cells in the kidneys, resulting in interstitial fibrosis and proximal tubular cell dysfunction [69]. Additionally, anemia is thought to decrease systemic vascular resistance and trigger compensatory glomerular hyperfiltration. While this aims at maintaining homeostasis, it mediates progressive renal damage and decline in glomerular filtration rate in the long run [70]. Iron overload has also been suggested as a prominent player in tubular and glomerular dysfunction. End-stage kidney disease is a possible end-result of anemia and iron overload-mediated kidney damage [71].

## 4. General Management

We base this section mostly on the TIF 2013 management guidelines, [11]. Otherwise, guidelines for specific morbidities mainly follow the general practice in non-thalassemic populations, although some studies are under way to better understand the actual effect of the application of general guidelines in the NTDT population [72].

### 4.1. Transfusion Therapy

Although regular RBC transfusion therapy is not required in NTDT, it is occasionally needed during times of increased physiological stress such as severe infection, pregnancy, surgery, and precipitous hemoglobin drops [2]. Symptoms and complications, rather than absolute hemoglobin level, remain the best determinant of the need for RBC transfusion therapy in NTDT [11]. Poor growth development in pre-adolescence, as well as some morbidities previously described, may indicate the need for regular RBC transfusion therapy. Some NTDT patients reach a point where regular transfusions become essential to maintain hemoglobin levels appropriate to sustain activity, growth, and development and to prevent further skeletal deformities [5].

Although definitive evidence is lacking, frequent RBC transfusion therapy is linked to reduced NTDT complications, including leg ulcers, thrombotic events, pulmonary hypertension, silent brain infarcts, and extramedullary hematopoietic pseudotumors [9,31,43,58,73]. Besides preventing hypoxia-related complications, RBC transfusion therapy is thought to improve coagulation defects and decrease the need for splenectomy [74]. Alternatively, RBC transfusion therapy is associated with worsening of iron overload and cardiac dysfunction [12]. There is a risk of alloimmunization with RBC transfusion therapy, especially in minimally transfused, pregnant, or splenectomized NTDT patients, but this risk can be mitigated through full phenotypic blood matching [5].

### 4.2. Splenectomy

Splenectomy was commonly pursued in NTDT to help maintain higher hemoglobin levels. In the light of increased morbidity with splenectomy, clinical practice has gradually shifted to restricting it to very specific indications such as poor growth and development, hypersplenism with symptomatic leukopenia and or thrombocytopenia, or symptomatic massive splenomegaly. Splenectomy is accompanied by an increased risk of severe infections, especially life-threatening post-splenectomy sepsis. Patients who will be splenectomized must receive the pneumococcal 23-valent polysaccharide vaccine, *Haemophilus influenzae* vaccine, and the meningococcal polysaccharide vaccine at least 2 weeks before the procedure [11]. The spleen scavenges procoagulant RBCs and platelets; therefore, splenectomy predisposes to hypercoagulability and accompanying concomitant increase in the incidence of PHT, silent cerebral infarcts, venous thromboembolism, and leg ulcers [5].

### 4.3. Hydroxyurea Therapy

Hydroxyurea induces *γ*-globin chain production, which combines with the excessive *α*-chains to produce HbF, thus alleviating the *α*/*β* globin chain imbalance and subsequently improving ineffective erythropoiesis [61]. Data from observational studies including OPTIMAL CARE suggests that hydroxyurea therapy is associated with lower rates of extramedullary hematopoiesis, osteoporosis, leg ulcers, hypothyroidism, and PHT [9]. Direct prospective evidence is still lacking and the clinical benefit of hydroxyurea therapy has not been systemically established [11]. Several non-randomized and retrospective studies have evaluated HbF inducers in NTDT and considered it to be safe and clinically effective. An increase in hemoglobin level by at least 1 g/dL at 6 months of therapy is considered to be an adequate response. Patients should be evaluated periodically afterwards to ensure benefit is maintained and to detect adverse effects including rashes, alopecia, gastrointestinal disturbances, and myelotoxicity [61].

### 4.4. Iron Chelation

A cut-off LIC value of >5 mg/g dw has been shown to be associated with significantly increased risk of several NTDT morbidities. Any increase above 5 mg/g dw by 1 mg/g dw is independently associated with increased risk of PHT, hypothyroidism, hypogonadism, osteoporosis, and thrombosis [62,75]. It is recommended to assess LIC using MRI T2*, R2, or R2* pulse sequences annually, in addition to serum ferritin level assessment every 3 months [11]. An interesting relationship between LIC and serum ferritin has been verified and used to guide iron chelation therapy in patients 10 years of age or older especially in areas where MRI is not available. An LIC of >5 mg/g dw correlated well with a serum ferritin of >800 ng/mL, defined as the level at which iron chelation therapy should be initiated in NTDT. An LIC of <3 gm/g dw correlated well with a serum ferritin of <300 ng/mL defined as the level at which discontinuing iron chelation in NTDT would be safe [76]. The drugs currently available for clinical use for iron chelation include oral deferiprone in solution or tablet form, deferoxamine in subcutaneous or intravenous injection, and oral deferasirox in dispersible tablet (DT) or film-coated tablet (FCT) form. While all three of these drugs have proven their effectiveness as iron chelators in TDT patients, deferasirox remains the only drug that has received Food and Drug Administration (FDA) and European Medicines Agency (EMA) approval for use in NTDT patients, mostly based on the results extracted and published from the THALASSA trial [77].

#### 4.4.1. Deferasirox

Based on the results of a placebo-controlled trial, deferasirox is the only iron chelating agent clinically proven to reduce iron loading in NTDT patients. Initiation of therapy with deferasirox is recommended at an LIC of ≥5 mg/g dw and at an initial dose of 10 mg/kg/day. Once LIC drops below 3 mg/g dw, treatment should be interrupted. LIC measurement is to be monitored every 6–12 months and serum ferritin every 3 months [61,78].

The most common side effects of deferasirox therapy include gastrointestinal disturbances, rash, increased hepatic transaminases, and elevated creatinine. Some of the rare adverse effects include gastrointestinal hemorrhage and severe renal and hepatic impairment. Side effects are most commonly encountered at higher doses of 25–35 mg/kg/day [79]. Patients on deferasirox therapy should be followed monthly with serum creatinine and urine protein/creatinine ratio. Increase in serum creatinine and proteinuria may necessitate dose reduction or treatment interruption. Monthly follow up of hepatic transaminases is also recommended. Additionally, contraception is recommended while on deferasirox. Yearly ophthalmic examination and audiometry testing are recommended after starting therapy [77,79,80,81].

#### 4.4.2. Deferiprone

Deferiprone has not been extensively studied as deferasirox in NTDT. It lacks support of large randomized controlled trials; however, results from single-arm, open-label trials and a recent randomized controlled showed significant decrease in serum ferritin and LIC. Adverse effects include agranulocytosis, neutropenia, arthralgias, elevated hepatic enzymes, and gastrointestinal disturbances [82,83,84].

#### 4.4.3. Deferoxamine

Studies of deferoxamine in NTDT have shown an increase in urinary iron excretion and a decrease in serum ferritin [85]. Robust evidence of the clinical efficacy of deferoxamine in NTDT is lacking. Significant adverse effects associated with deferoxamine therapy include skeletal abnormalities, growth retardation, ocular disturbances, local infusion site reactions, and auditory disturbances [86,87].

### 4.5. Hematopoietic Stem Cell Transplantation

Until now, hematopoietic stem cell transplantation (HSCT) to replace mutant cell lines remains the only curative remedy for all thalassemic disorders, with chances as high as 80% in HLA-matched sibling donor transplants [88]. Gene therapy with lentivirus-mediated gene transfer of healthy genes to affected cell lines and gene editing are also being studied [89,90].

## 5. Future Interventions

In the past decade, several therapeutic options have emerged for patients with *β*-thalassemia. These advances aim at improving iron dysregulation, globin-chain imbalance, and/or ineffective erythropoiesis.

### 5.1. Improving Iron Dysregulation

Minihepcidins, long-acting hepcidin analogs, are currently being studied in clinical trials with hypothesized outcomes including decreasing iron absorption levels from the gastrointestinal tract, regulating iron handling by macrophages, increasing hemoglobin concentration, and reducing spleen size [91]. An alternative method for hepcidin levels is currently being investigated using anti-sense oligonucleotides and small interfering RNA to block the hepcidin-degrading effect of *TMPRSS6*, a transmembrane protease [90,92]. Genetic ablation of *TMPRSS6* also improved ineffective erythropoiesis and decreased splenomegaly in *β*-TI, without a concomitant decrease in erythropoietin production [23]. Normalization of RBC survival is a significant component of the effects of *TMPRSS6* inhibition on both hemoglobin and spleen size. 

### 5.2. Correcting Globin-Chain Imbalance

Gene therapy is a promising treatment modality in the management of thalassemia. Some recent studies have described the long-term correction of murine models of human *β*-thalassemia and sickle cell anemia by lentivirus-mediated gene transfer [93,94]. The emergence of gene editing technology, whether by direct correction of genetic mutations in the endogenous DNA of the cell or by disruption of specific DNA sequences in the genome, offers a new approach for treating *β*-thalassemia. This is facilitated by site specific double strand breaks which can be induced with zinc finger nucleases, transcription activator-like effector nucleases, meganucleases, and, more recently, clustered regularly interspaced short palindromic repeats (CRISPR)/Cas9 system [89]. Other alternatives that could allow more efficient and immediate treatment of *β*-thalassemia with genome editing include the disruption of factors that silence the *γ*-globin genes, such as *BCL11A* or *γ*-globin repressive elements within the *β*-globin gene locus [95,96].

### 5.3. Improving Ineffective Erythropoiesis

Recent studies have elucidated the roles of Janus Kinase 2 (JAK2) and the transforming growth factor (TGF)-*β* superfamily in the control of erythropoiesis. Binding of erythropoietin to its cell membrane receptor activates the cytoplasmic JAK2, which in turn activates multiple signal transduction pathways to increase proliferation, differentiation, and survival of erythroid progenitors. JAK2 is the only intracellular signal transductor of erythropoietin and is, therefore, a potential target to treat conditions caused by disordered and ineffective erythropoiesis [15]. JAK2 inhibitors, such as ruxolitinib, have shown promise for amelioration of average hemoglobin concentration and potential spleen size reduction in patients with TDT; the results of such trials in TDT may lay the basis for studies in NTDT especially in patients with enlarged spleens [97].

Two activin receptor fusion proteins, sotatercept (ACE-011) and luspatercept (ACE-536), have been developed for the treatment of conditions caused by ineffective erythropoiesis, including *β*-thalassemia. These recombinant proteins bind to select TGF-*β* superfamily ligands that regulate late-stage erythropoiesis [98]. Thus, the mechanisms of action of sotatercept and luspatercept are distinct from erythropoiesis-stimulating agents and erythropoietin, which act on earlier stages of erythropoiesis. Available data indicate that luspatercept was generally well tolerated and had a favorable safety profile. It reduced transfusion requirements and liver iron concentration among patients with TDT and increased hemoglobin levels, reduced liver iron concentration, and improved quality of life among those with NTDT [99]. Current ongoing clinical trials are looking at the efficacy and safety of luspatercept in TDT and NTDT patients.

## 6. Conclusions

NTDT has a unique morbidity profile that generally starts manifesting later in life, when compared to TDT. The complications seen in NTDT tend to follow the natural progression of the disease since regular RBC transfusion therapy is not indicated. In order to better understand each complication and its appropriate management, it is important to find correlations with the pathophysiological hallmarks of the disease. Furthermore, the dramatic improvement in therapeutic practices and the observed survival benefit in NTDT over the past decades uncover newer challenges associated with more complicated comorbidities from long standing anemia, tissue hypoxia, and possibly iron overload. Timely medical intervention remains of critical importance to curtail the development of long-term complications that may reach the point of irreversibility.

## Figures and Tables

**Table 1:** Several *β*-Thalassemia variants that can present as non-transfusion dependent thalassemia.

Homozygosity for mild forms of *β*^+^ thalassemia
Compound heterozygosity for *β*^+^/*β*^0^ thalassemia
Compound heterozygosity for *β*-thalassemia and another *β* chain variant (e.g., *β*-thal/hemoglobin HbE)
Coinheritance of homozygous *β*-thalassemia and hereditary persistence of fetal Hemoglobin [HPFH])
Coinheritance of homozygous *β*^+^ thalassemia with *α*-thalassemia (e.g., *β*^+^/*β*^+^ with −*α*/−*α*, −−/*αα*, −*α*/*αα*, or −−/−*α*)
Coinheritance of heterozygous *β*-thalassemia and triplicated or quadruplicated *α* genes (eg, *αα*/*ααα* or *αα*/*ααα*)
Dominant forms of *β*-thalassemia

*β*^0^ thalassemia: no production of *β* chains; *β*^+^ thalassemia: reduced production of *β* chains (may be mild, moderate or severely reduced).

## Abbreviations

NTDT	Non-Transfusion Dependent Thalassemia
IOL	Iron Overload
LV	Left Ventricle
TDT	Transfusion Dependent Thalassemia
TMPRSS-6	Transmembrane Protease Serine-6
PHT	Pulmonary Hypertension
TI	Thalassemia Intermedia
CO	Cardiac Output
RBC	Red Blood Cell
PVR	Pulmonary Vascular Resistance
Hb	Hemoglobin
HCC	Hepatocellular Carcinoma
HbF	Fetal Hemoglobin
HCV	Hepatitis C Virus
HBV	Hepatitis B Virus
TIF	Thalassemia International Federation
LIC	Liver Iron Concentration
BMD	Bone Mineral Density
MRI	Magnetic Resonance Imaging
GI	Gastrointestinal
dw	Dry Weight
DNA	Deoxyribonucleic Acid
JAK2	Janus Kinase-2
CRISPR	Clustered Regularly Interspaced Short Palindromic Repeats
